# Microbial life at high salt concentrations: phylogenetic and metabolic diversity

**DOI:** 10.1186/1746-1448-4-2

**Published:** 2008-04-15

**Authors:** Aharon Oren

**Affiliations:** 1 Department of Plant and Environmental Sciences, The Institute of Life Sciences, and the Moshe Shilo Minerva Center for Marine Biogeochemistry, The Hebrew University of Jerusalem, Jerusalem 91904, Israel

## Abstract

Halophiles are found in all three domains of life. Within the Bacteria we know halophiles within the phyla *Cyanobacteria*, *Proteobacteria*, *Firmicutes*, *Actinobacteria*, *Spirochaetes*, and *Bacteroidetes*. Within the Archaea the most salt-requiring microorganisms are found in the class *Halobacteria*. *Halobacterium *and most of its relatives require over 100–150 g/l salt for growth and structural stability. Also within the order *Methanococci *we encounter halophilic species. Halophiles and non-halophilic relatives are often found together in the phylogenetic tree, and many genera, families and orders have representatives with greatly different salt requirement and tolerance. A few phylogenetically coherent groups consist of halophiles only: the order *Halobacteriales*, family *Halobacteriaceae *(*Euryarchaeota*) and the anaerobic fermentative bacteria of the order *Halanaerobiales *(*Firmicutes*). The family *Halomonadaceae *(*Gammaproteobacteria*) almost exclusively contains halophiles. Halophilic microorganisms use two strategies to balance their cytoplasm osmotically with their medium. The first involves accumulation of molar concentrations of KCl. This strategy requires adaptation of the intracellular enzymatic machinery, as proteins should maintain their proper conformation and activity at near-saturating salt concentrations. The proteome of such organisms is highly acidic, and most proteins denature when suspended in low salt. Such microorganisms generally cannot survive in low salt media. The second strategy is to exclude salt from the cytoplasm and to synthesize and/or accumulate organic 'compatible' solutes that do not interfere with enzymatic activity. Few adaptations of the cells' proteome are needed, and organisms using the 'organic-solutes-in strategy' often adapt to a surprisingly broad salt concentration range. Most halophilic Bacteria, but also the halophilic methanogenic Archaea use such organic solutes. A variety of such solutes are known, including glycine betaine, ectoine and other amino acid derivatives, sugars and sugar alcohols. The 'high-salt-in strategy' is not limited to the *Halobacteriaceae*. The *Halanaerobiales *(*Firmicutes*) also accumulate salt rather than organic solutes. A third, phylogenetically unrelated organism accumulates KCl: the red extremely halophilic *Salinibacter *(*Bacteroidetes*), recently isolated from saltern crystallizer brines. Analysis of its genome showed many points of resemblance with the *Halobacteriaceae*, probably resulting from extensive horizontal gene transfer. The case of *Salinibacter *shows that more unusual types of halophiles may be waiting to be discovered.

## Introduction

At the symposium on 'General & Applied Aspects of Halophilic Microorganisms', held in Alicante, Spain in September 1989, Hans Trüper presented a lecture entitled: "Halophily, taxonomy, phylogeny and nomenclature". The talk summarized different aspects of the taxonomy of halophilic microorganisms, the phylogenetic position of the different types of halophiles within the microbial world, and the physiology of the diverse groups of extreme and moderate halophiles. An attempt was made to detect consistent patterns and correlations between the phylogeny of halophilic microorganisms and the ways they cope with the high salt concentrations in the environment in which they live. A summary of this talk was published three years later in the proceedings volume of the Alicante meeting [1], and there Prof. Trüper and his coworkers presented a critical analysis of the data. They noted a few regular patterns that link the physiological properties of the halophilic organisms with their phylogenetic position. Four such general observations were presented:

(1) "All eubacteria that gain energy from photosynthesis or oxygen-respiration and are capable of haloadaptation, are able to accumulate and/or produce compatible solutes."

(2) "Archaeobacteria and anaerobic fermenting eubacteria are incapable of synthesizing organic compatible solutes."

(3) "Most eubacteria that can grow in non-complex media are capable of ectoine biosynthesis."

(4) "Extreme halophily in non-fermenting eubacteria is usually accompanied by glycine betaine biosynthesis. The complete de novo synthesis of betaine from carbon dioxide or simple organic carbon compounds has only been proven for cyanobacteria, *Ectothiorhodospira *species and *Actinopolyspora halophila*."

In the eighteen years that have passed since the Alicante meeting our understanding of the salt-loving microbial world has greatly increased. A large number of new halophilic organisms have been discovered and described, including organisms phylogenetically unaffiliated to previously known halophilic or halotolerant microorganisms. We have also obtained a deeper insight into the physiological mechanisms used by different types of halophiles to adapt to life in hypersaline environments.

Based on the key words in the title of Prof. Trüper's talk – "halophily", "taxonomy", "phylogeny" and "nomenclature", this paper first presents an overview of the novel information, followed by a reevaluation of the four above-cited general observations made in 1989.

### Halophily and the phylogenetic tree of life

Salt dependence and salt tolerance are phenotypic characteristics generally included in the 'polyphasic' characterization of newly discovered microorganisms toward their description as new taxa with new names and the determination of their position within the microbial taxonomy. There is no sharp definition of the term 'halophilic' – 'salt-loving'. Some use the term for all organisms that require some level of salt for growth, including concentrations around 35 g/l as found in seawater. This is also reflected in prokaryote nomenclature: there are quite a few bacteria with the specific epithet '*halophilus*' (-*a*, -*um*) that are of marine origin and do not tolerate salt concentrations much above those of seawater. Examples are *Aestuariibacter halophilus*, *Algoriphagus halophilus *(*Hongiella halophila*), *Arcobacter halophilus*, *Microbacterium halophilum*, and *Terribacillus halophilus*. The use of the specific epithet *halophilus *to describe marine microorganisms should be discouraged, and it is recommended to reserve this name for true halophiles adapted to life in hypersaline conditions.

A survey of the salt relationships – the minimum salt concentration required for growth, the salinity optimum, and the upper salt limit tolerated – within the microbial world shows a continuum of properties, which makes it nearly impossible to define by sharp boundaries what a halophile is. Moreover, the minimum, optimum and maximum salt concentrations often depend on the medium composition and growth temperature. The most widely used definitions were formulated thirty years ago by Donn Kushner [2] who distinguished different categories: extreme halophiles (growing best in media containing 2.5–5.2 M salt), borderline extreme halophiles (growing best is media containing 1.5–4.0 M salt), moderate halophiles (growing best in media containing 0.5–2.5 M salt), and halotolerant microorganisms that do not show an absolute requirement for salt for growth but grow well up to often very high salt concentrations (considered extremely halotolerant if the growth range extends above 2.5 M salt). These and similar definitions have proved valuable in the classification of microorganisms according to their relationship to salt [3-5]. For the discussions below a simpler operative definition of what a halophile is will suffice: microorganisms that grow optimally at salt concentrations of 50 g/l (equivalent to 0.85 M NaCl) or higher, and tolerate 100 g/l salt (equivalent to 1.7 M NaCl) at least.

**Figure 1:**
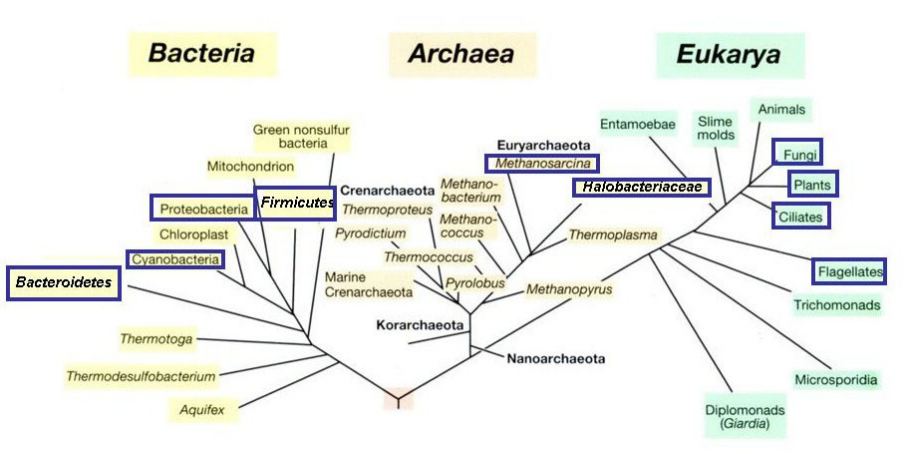
**The universal phylogenetic tree of life as based on small subunit rRNA gene sequences, and the distribution of halophilic microorganisms within the tree.** Groups marked with blue boxes contain at least one halophilic representative (e.g the *Bacteroidetes*, of which *Salinibacter ruber *is the sole halophilic member described to date); others such as the *Halobacteriales *consist entirely of halophiles. The tree is based on Fig. 11.16 in Madigan and Martinko, 2006 (78).

When examining the distribution of halophiles, as based on the above definition, within the small subunit rRNA sequence-based phylogenetic tree of life, it is clear that halophiles are found in all three kingdoms: Archaea, Bacteria, and Eukarya (Fig. 1) [3,4,6]. The figure indicates which groups of microorganisms contain halophilic representatives. Groups marked as such do not necessarily consist solely of halophiles. The opposite is true: there are only a few phylogenetically consistent groups that are composed entirely of halophiles (see below). In most cases halophiles and non-halophilic relatives are found together in the phylogenetic tree, and many genera, families and orders have representatives with greatly different salt requirement and tolerance. Within the Archaea we find the most salt-requiring microorganisms in the order *Halobacteriales*, which contains a single family, the *Halobacteriaceae*. *Halobacterium *and most of its relatives require over 100–150 g/l salt for growth and structural stability. Also within the class *Methanothermea *(*Methanococci*), order *Methanosarcinales *we encounter halophilic or highly halotolerant representatives (genera *Methanohalophilus*, *Methanohalobium*). All these belong to the phylum *Euryarchaeota*; no halophilic representatives have yet been identified within the *Crenarchaeota*. Halophily is widespread in the bacterial kingdom: we know halophiles within the phyla *Cyanobacteria*, *Proteobacteria*, *Firmicutes*, *Actinobacteria*, *Spirochaetes*, and *Bacteroidetes*.

With a few notable exceptions, the eukaryotic microorganisms form a sadly neglected group as far as the study of their distribution in high-salt environments and their physiological adaptation to life at high salt concentrations is concerned. The unicellular green algae of the genus *Dunaliella *have been investigated in-depth, as they are the main or sole primary producers in many hypersaline environments, they have become a popular model system for the study of salt adaptation by using organic osmotic 'compatible' solutes, and they have also found biotechnological applications [7]. The occurrence of the brine shrimp (*Artemia salina*, *Artemia franciscana*) in salt lakes is also well known, but the discussion of its biology is outside the scope of this review. The Fungi, long neglected in halophile research, contain a number of representatives that by all criteria are true halophiles, both by their absolute requirement for high salt and by their ability to grow up to salt concentrations approaching saturation. Examples are the meristematic fungus *Trimmatostroma salinum *[8] and the black yeast *Hortaea werneckii *[9], organisms indigenous to saltern brines and other hypersaline environments. Even more severely neglected is the study of flagellate, ciliate and amoeboid protozoa that live in high-salt environments. The existence of such organisms has been documented already long ago [[10,11]; for an overview of the early literature see [12]), but only recently has the study of truly halophilic heterotrophic nanoflagellates taken off. Different types of halophilic flagellates were found in Korean saltern ponds [13], and two species have thus far been characterized in-depth. The first is *Pleurostomum flabellatum*, an organism known already since the 1930s. An isolate from a saltern pond with 313 g/l salt has its optimum at 300 g/l salt and does not grow below 150–200 g/l [14]. Another bacteriovorous heterotrophic nanoflagellate isolated from a 300 g/l saltern pond is *Halocafetaria seosinensis*, which grows optimally at 150 g/l salt, does not grow below 75 g/l, and tolerates concentrations above 350 g/l total dissolved salts [15].

### The *Halobacteriales*, the *Halomonadaceae*, and the *Halanaerobiales*: three phylogenetically coherent groups of halophiles

Within the small subunit rRNA gene sequence-based tree of life we find three groups of prokaryotes that are both phylogenetically and physiologically coherent and consist entirely or almost entirely of halophiles. Within the *Euryarchaeota *we encounter the order *Halobacteriales *with a single family, the *Halobacteriaceae *[16]. In the bacterial kingdom, the family *Halomonadaceae *(class *Gammaproteobacteria*, order *Oceanospirillales*) predominantly contains halophiles [17]. Members of the *Halobacteriaceae *and the *Halomonadaceae *are aerobic heterotrophs, some of which have a limited potential for anaerobic growth. The third phylogenetically coherent group contains the anaerobic fermentative bacteria of the order *Halanaerobiales *(*Firmicutes*, families *Halanaerobiaceae *and *Halobacteroidaceae*) [18].

**Figure 2:**
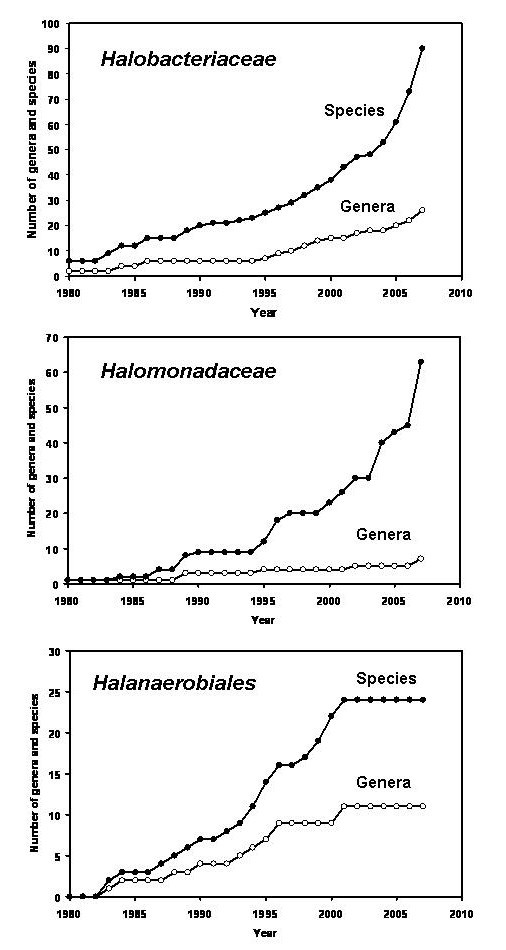
Numbers of different genera and species within the family *Halobacteriaceae*, the family *Halomonadaceae*, and the order *Halanaerobiales*, whose names have been validly published, 1980–2007.

Our understanding of the phylogenetic and physiological diversity within the *Halobacteriaceae *and the *Halomonadaceae *has greatly increased in the past two decades. Extensive programs aimed at the sampling of salt lakes and other saline and hypersaline environments in different countries have led to the characterization and taxonomic description of many new species, so that the number of species names within these two families has increased exponentially. The 1974 edition of 'Bergey's Manual of Determinative Bacteriology' listed 2 genera and 3 species within the family *Halobacteriaceae *[19]. The next edition of the handbook, published in 1989 under the new name 'Bergey's Manual of Systematic Bacteriology', already had 6 genera and 11 species [20]. The numbers of genera and species increased to 14 and 34, respectively in the 2001 edition [21], and to date (March 2008) the names of no less than 26 genera and 91 different species of *Halobacteriaceae *have been validly published under the rules of the International Code of Nomenclature of Prokaryotes (Fig. 2, upper panel). With descriptions of eight additional new species listed as 'in press' in the International Journal of Systematic and Evolutionary Microbiology (as of March 2, 2008), the current trend is expected to continue at least for some more time.

Most species of *Halobacteriaceae *are true extreme halophiles according to Kushner's definition [2]. However, analysis of 16S rRNA genes amplified from environments with salt concentrations below 100 g/l shows that the lower salinity boundary that allows development of archaeal halophiles may be substantially lower than that of *Halobacterium salinarum*, *Haloarcula marismortui*, and other well-studied members of the group [22]. Descriptions of isolates phylogenetically affiliated with the *Halobacteriaceae *but with a relatively low salt requirement also started to appear in the literature. A saline spring in Oklahoma whose waters contain 7–10 g/l salt, a concentration far too low to support growth of most members of the *Halobacteriaceae*, recently yielded a novel species of *Haloferax*, *Haloferax sulfurifontis *[23] and two species belonging to new genera:*Haladaptatus paucihalophilus *(the specific epithet signifying its low salt requirement) [24] and *Halosarcina pallida *[25]. The lower salinity boundary of these species for growth is 60, 47, and 76 g/l, respectively, but the optimal salt concentrations and the maximal salinity tolerated by these isolates are within the high ranges normally encountered for members of the family. *Halobacterium salinarum *and most other species of *Halobacteriaceae *lyse when the salt concentration is lowered below 100 g/l. However, some of its relatives have a remarkable ability to withstand exposure to low salt concentrations. Out of 131 isolates of *Halobacteriaceae *obtained from Spanish saltern ponds, 49 did not lyse in 20 g/l salt and 13 even survived exposure to 10 g/l salt [26]. It was recently shown that isolates obtained from Japanese salterns and affiliated with the genera *Halococcus*, *Halogeometricum *and *Haladaptatus *may survive periods of 1–9 days in salt solutions of seawater concentration [27]. *Haloferax sulfurifontis *survived incubation for 24 h at 10–20 g/l salt and 72 h in 30 g/l salt [23], and *Haladaptatus paucihalophilus *cells even retained their viability after having been suspended for two weeks in distilled water [24]. The sediments of a British salt marsh yielded several isolates of *Halobacteriaceae*, many of which closely related to *Haladaptatus paucihalophilus*, which grow slowly at seawater salinity [28].

It must be realized that there is no well-based species concept for the prokaryotes, and the same is therefore true for the halophilic Archaea. The above-presented statistics of the number of species described are all based on some more or less arbitrary ideas of how to define a species and when to decide whether a new isolate may belong to an already known species or should be described as a novel one. A recent evaluation of a large number of isolates of *Halobacteriaceae *from saltern ponds in Spain and Algeria using multilocus sequence analysis shows that, although clusters can be defined by concatenation of multiple marker sequences, barriers to exchange between them are leaky. No nonarbitrary way to circumscribe 'species' is therefore likely to emerge for the *Halobacteriaceae *in the near future [29].

A dramatic increase in the number of descriptions of new species in recent years can also be observed for the second phylogenetic group that consists (almost) entirely of halophiles: the family *Halomonadaceae *(*Gammaproteobacteria*). When *Halomonas elongata *was isolated in 1980 [30], few people predicted that it would be the first representative of a very large group of metabolically versatile moderate halophiles. The recognition that *Halomonas *and relatives deserve to be classified in a separate family came in 1988 [31], and the number of species and genera has been rising steadily since ([5,32,33]; Fig. 2, middle panel). Recently two new genera were added to the family, *Halotalea *and *Modicisalibacter *[34,35], bringing the census for March 2008 to 7 genera with 63 species whose names have been validly published. Out of these 63 species, 60 can be considered halophiles as based on the above definition. The three genera *Zymomonas*, *Carnimonas*, and *Cobetia *each contain presently one non-halophilic or marine species. As of March 2, 2008, four new species of the genus *Halomonas *were listed as 'in press' in the International Journal of Systematic and Evolutionary Microbiology.

The third phylogenetically coherent group consisting entirely of halophiles is the interesting but little studied order *Halanaerobiales*, families *Halanaerobiaceae *and *Halobacteroidaceae *(Low G+C branch of the *Firmicutes*) [18]. This group consists of obligate anaerobes, and most live by fermentation of sugars or (in a few cases only) amino acids. That the study of this group has to some extent been neglected in comparison with the other halophile branches is obvious from the number of new species described: the count for March 2008 was 11 genera and 24 species (Fig. 2, lower panel), the two most recently added genera and species – *Halonatronum saccharophilum *and *Selenihalanaerobacter shriftii *having been described in 2001. The latter species is of special interest as it makes a living by anaerobic respiration rather than by fermentation [36]. The family *Halanaerobiaceae *also contains a thermophilic representative: *Halothermothrix orenii*, an organism isolated from a hypersaline lake in Tunisia that combines anaerobic life at high salt (optimum 100 g/l, tolerating up to 200 g/l salt) with a high temperature optimum (60°C, growing up to 68°C) [37].

### Mechanisms of adaptation of halophilic microorganisms to life at high salt

Trüper's four 'postulates', as presented at the Alicante symposium [1], deal with the presence, distribution and biosynthesis of organic osmotic solutes, and they mention specific compounds such as ectoine and glycine betaine.

A basic property of all halophilic microorganisms is the fact that their cytoplasm has to be at least isoosmotic with their surrounding medium. Biological membranes are permeable to water, and active energy-dependent inward transport of water to compensate for water lost by osmotic processes is energetically not feasible. Moreover, cells that keep a turgor need even to maintain their intracellular osmotic pressure higher than that of their environment [38,39].

There are two fundamentally different strategies used by halophilic microorganisms to balance their cytoplasm osmotically with their medium. The first involves accumulation of molar concentrations of potassium and chloride. This strategy requires extensive adaptation of the intracellular enzymatic machinery to the presence of salt, as the proteins should maintain their proper conformation and activity at near-saturating salt concentrations [40]. The proteome of such organisms is highly acidic, and most proteins denature when suspended in low salt. Such 'high-salt-in strategy' microorganisms generally cannot survive in low salt media.

**Figure 3:**
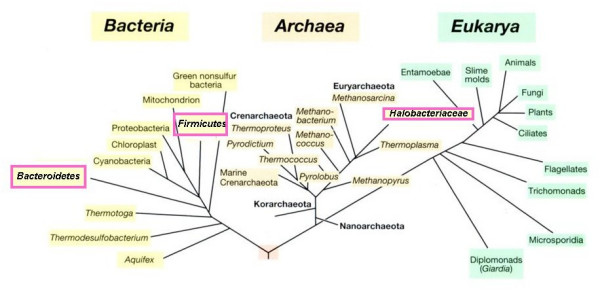
**Distribution within the phylogenetic tree of microorganisms accumulating KCl as their sole or main osmotic solute. **Groups marked with purple boxes contain at least one halophilic representative (e.g. *Salinibacter ruber *within the *Bacteroidetes*). The group of the *Firmicutes *contains both microorganisms that use KCl for osmotic balance (the order *Halanaerobiales *within the low G+C branch, consisting of anaerobic fermentative organisms) and different halophilic aerobes (*Halobacillus *spp. and others) that accumulate inorganic solutes. High intracellular KCl concentrations are also found in the methanogenic halophiles, but these accumulate organic solutes as well.

Although it can be calculated that the 'high-salt-in strategy' is energetically less costly to the cell than the biosynthesis of large amounts of organic osmotic solutes [41], this strategy is not widely used among the different phylogenetic and physiological groups of halophiles (Fig. 3). It is best known from the extremely halophilic Archaea of the family *Halobacteriaceae*, and species such as *Halobacterium salinarum *and *Haloarcula marismortui *have become popular model organisms to examine the implications that the maintenance of high intracellular KCl concentrations has on the life of a cell. Our understanding of the biology of the *Halobacteriaceae *has greatly increased in recent years thanks to the elucidation and analysis of the genome sequences of *Halobacterium *NRC-1 [42,43], *Haloarcula marismortui *[44], *Natronomonas pharaonis *[45], and *Halquadratum walsbyi *[46].

The strategy of salt adaptation is not limited to the aerobic halophilic Archaea. The anaerobic fermentative *Halanaerobiales *(Bacteria, *Firmicutes*) also use KCl rather than organic solutes to osmotically balance their cytoplasm, and they also have adapted their intracellular machinery to tolerate the presence of salt [18,47]. The third type of organism, phylogenetically unrelated with the above-mentioned two groups, in which the 'high-salt-in-strategy' was recently identified to occur, is the aerobic red extremely halophilic *Salinibacter ruber *(*Bacteroidetes*). The properties of this intriguing organism are discussed below in further depth.

**Figure 4:**
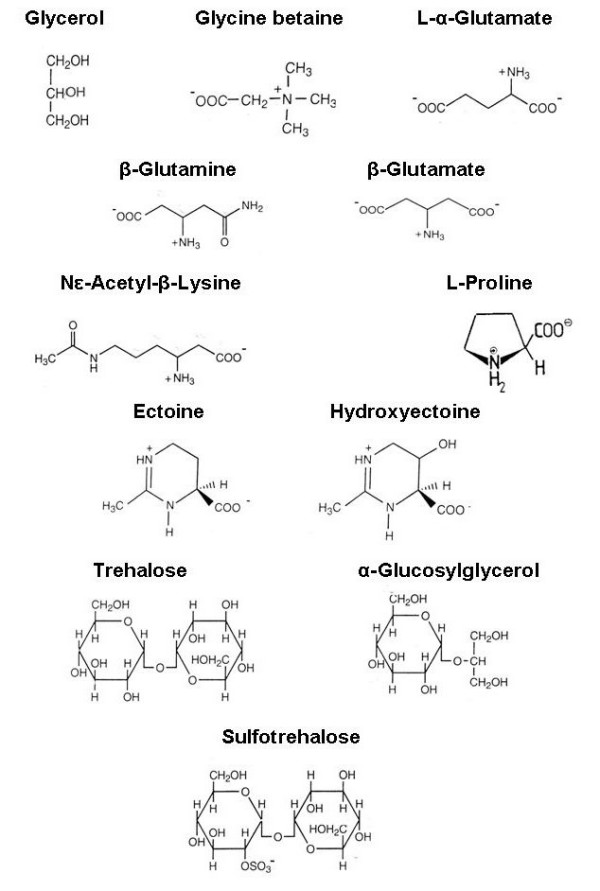
A selection of organic osmotic solutes found in halophilic and halotolerant prokaryotic and eukaryotic microorganisms.

Far more widespread in nature is the second strategy of haloadaptation, based on the biosynthesis and/or accumulation of organic osmotic solutes. Cells that use this strategy exclude salt from their cytoplasm as much as possible. The high concentrations of organic 'compatible' solutes do not greatly interfere with normal enzymatic activity. Few adaptations of the cells' proteome are therefore needed. Such organisms can often adapt to a surprisingly broad salt concentration range [5]. The list of organic compounds that have been shown to serve as osmotic solutes in halophilic microorganisms – prokaryotic as well as eukaryotic – is extensive. Most compatible solutes are based on amino acids and amino acid derivatives, sugars, or sugar alcohols [48-50]. Most are either uncharged or zwitterionic (Fig. 4).

**Figure 5:**
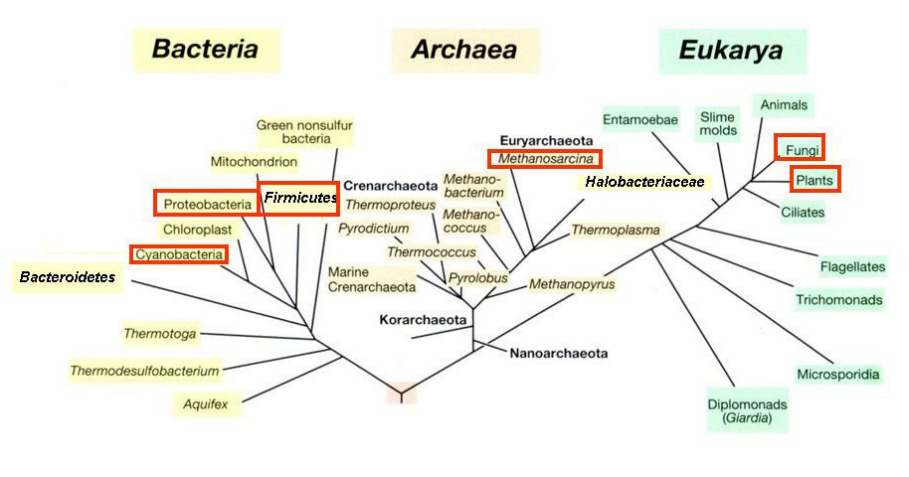
**Distribution within the phylogenetic tree of microorganisms accumulating organic solutes to provide osmotic balance.** Groups marked with red boxes contain at least some halophilic representatives in which de novo synthesis and/or accumulation of organic solutes has been demonstrated. The group of the *Firmicutes *contains both halophilic aerobes that accumulate organic solutes and anaerobic fermentative microorganisms (the order *Halanaerobiales*) that use KCl.

Use of the 'low-salt-in strategy' of haloadaptation with accumulation of organic osmotic solutes is widespread in the small subunit rRNA sequence-based phylogenetic tree of life (Fig. 5). Not all groups of halophiles have yet been examined for the occurrence and distribution of organic solutes. For example, no information is available on the composition and concentrations of intracellular solutes within the recently characterized extremely halophilic flagellate protozoa [14,15]. We do, however, have a quite complete picture of the distribution of organic solutes in most other groups of halophilic microorganisms. Glycerol and other polyols are widely used for osmotic adaptation in halophilic eukaryotic algae and fungi, but only seldom in the the prokaryotes, the finding of mannitol in *Pseudomonas putida *[51] being a notable exception. The occurrence of certain other solutes in halophilic and halotolerant prokaryotes is also often correlated with their position in the phylogenetic tree of life.

Within the domain Archaea we find a few solutes not yet detected elsewhere within the tree [49]. Halophilic methanogens such as the *Methanohalophilus *species contain, in addition to glycine betaine found widespread in nature, β-amino acids and derivatives that are rarely found in other groups: β-glutamine, β-glutamate, and Nε-acetyl-β-lysine ([49,52]; for a report on the occurrence of β-glutamate in the domain Bacteria see [53]). Sulfotrehalose has thus far been found only in a few alkaliphilic members of the *Halobacteriaceae*; it is accumulated in substantial concentrations (up to 1 M) in addition to KCl which serves as the main osmotic solute like in their neutrophilic relatives [54].

In the kingdom Bacteria the largest diversity of organic solutes is detected. Some compounds appear to be used by a wide range of organisms. Glycine betaine is readily used as osmotic solute by many different types [55], but only when it can be taken up from the medium. Relatively few prokaryotes are capable of de novo synthesis of the compound. Notably these are different types of phototrophs: halophilic cyanobacteria as well as anoxygenic phototrophs such as *Halorhodospira *spp. Occurrence of glycine betaine biosynthesis in the heterotrophic *Actinopolyspora halophila *(*Actinobacteria*) has been established long ago. In recent years the list of non-phototrophic prokaryotes capable of de novo production of glycine betaine as regulated by the extracellular salt concentration has grown. The methanogenic archaeon *Methanohalophilus portucalensis *synthesizes glycine betaine by reductive methylation of glycine that is generated from serine [50,56-59]. In contrast, the far less halotolerant *Methanosarcina mazei *does not produce glycine betaine, but accumulates the compound from the medium when exposed to salt stress. Recently evidence was obtained for de novo biosynthesis of glycine betaine also in the haloalkaliphilic chemoautotrophic sulfur-oxidizing *Proteobacteria Thioalkalibacter *and *Thioalkalivibrio *[60,61], but no information is as yet available about the biosynthetic pathway used and its regulation.

Far more abundant than glycine betaine as compatible solute in the domain Bacteria are the cyclic amino acid derivatives ectoine and hydroxyectoine, originally discovered in anoxygenic phototrophs of the *Ectothiorhodospira *– *Halospira *group [48]. Ectoine is now known to be synthesized by many aerobic heterotrophic bacteria [5,49]. Ectoine was also recently identified as a major osmotic solute in halophilic methanotrophs and methylotrophs [62,63].

The list of organic compatible solutes that have been identified is long, and Fig. 4 gives a representative selection only. Some compounds are widespread along the phylogenetic tree, while others appear to be limited to selected groups. For example, glucosylglycerol is almost exclusively found in moderately halophilic or highly halotolerant cyanobacteria, but has been detected in some pseudomonads as well [64,65]; Nε-acetyl-α-lysine and Nδ-acetylornithine have thus far been detected only in aerobic members of the *Firmicutes*. Another compound of interest is proline, found especially in the *Firmicutes *[66,67], but also present in halophilic/halotolerant diatoms [68]. A special biosynthetic pathway for the production of proline for osmotic purposes has been detected in *Bacillus subtilis *[69].

Use of organic osmotic solutes, whose intracellular concentrations can be regulated in accordance with the external salt concentration, provides microorganisms with a large degree of flexibility and the possibility to adapt to a wide range of salt concentrations [5,48]. However, energetically the production of massive amounts of such solutes can be costly. In addition, energy is still needed to prevent salts from reaching the cytoplasm. The high energy cost involved is especially relevant for microorganisms that obtain small amounts of energy from the dissimilatory processes they perform. A calorimetric study with *Halomonas elongata*, a bacterium that produces ectoine as its main compatible solute, shows that the organism has optimized its metabolism to minimize the energetic cost of osmotic adaptation [70]. It is well possible that the upper salinity at which different metabolic types of prokaryotes are found in nature depends to a large extent on the balance between the amount of energy that is available to the cells and the cost of production of organic solutes needed to provide osmotic balance [41].

### The case of *Salinibacter*, a member of the Bacteria with archaeal characteristics

Until recently it was generally accepted that halophilic members of the bacterial domain use organic solutes for osmotic balance. The sole exception known was that of the *Halanaerobiales*, a group of fermentative halophiles phylogenetically affiliated with the low G+C branch of the *Firmicutes*. It was also clear why the 'high-salt-in strategy' would be the preferred or possibly the only feasible mode of haloadaptation in this group: only very little energy is obtained in the fermentation pathways that provide ATP to these organisms, and accordingly the production of massive amounts of organic compatible solutes would leave insufficient energy for other cellular functions [41].

The discovery of *Salinibacter *and the analysis of its properties (Table 1) now require a change in our views. *Salinibacter ruber *is a red extreme halophile, phylogenetically belonging to the *Bacteroidetes *branch of the Bacteria, and it coexists with Archaea of the family *Halobacteriaceae *in NaCl-saturated saltern crystallizer ponds and in other hypersaline environments at or approaching halite saturation. Its existence was first recognized in the late 1990s based on the molecular characterization of the microbial community in Spanish saltern crystallizer ponds [71]. The isolation of the organism soon followed [72-74].

**Table 1:** A comparison of the properties of

	*Salinibacter*	*Halobacteriaceae*
Salt requirement	>150 g/l	>150 g/l in most species
Salt optimum	150–300 g/l	200–250 g/l in most species
G+C % in DNA	66.2	59–71; 46.9 in *Haloquadratum walsbyi*
Osmotic solute	KCl	KCl
Enzymes	Salt-requiring and salt-tolerant	Mostly salt-requiring
Lipids	Bacterial, including unusual sulfonolipids	Archaeal
Carotenoid pigments	C-40 substituted carotenoid ('salinixanthin')	C-50 bacterioruberins
Retinal pigments	Bacteriorhodopsin ('xanthorhodopsin'), halorhodopsin (not yet proven to be functional), sensory rhodopsins	Bacteriorhodopsin, halorhodopsin, sensory rhodopsins (in many but not in all species)

The affiliation of *Salinibacter *with the *Bacteria *appears not only from the sequence of its 16S rRNA gene, but also from the nature of the lipids in its membrane – bacterial lipids, in part of an unusual type, but with conventional fatty acids rather than the archaeal phytanyl-based hydrophobic chains [75]. The sensitivity of *Salinibacter *to antibiotics points to the presence of peptidoglycan in its cell wall. It was therefore expected that, like all other known halophilic and halotolerant aerobic Bacteria, *Salinibacter *would contain an organic solute or a cocktail of such solutes. However, no significant concentrations of organic solutes were detected. Instead, the cells contain molar concentrations of KCl [76], and its proteome is highly acidic, nearly as much so as the proteome of *Halobacterium *[77]. Analysis of the genome of *S. ruber *showed many additional common properties with the extremely halophilic Archaea. Extensive gene exchange may have occurred between *Salinibacter *and the *Halobacteriaceae*, which share the same environment and have been subject to the same environmental stress factors throughout their evolutionary history [77].

### Final comments

Based on all the new information that has become available in the past two decades we can now reevaluate the four 'postulates' presented by Trüper at the Alicante 1989 halophile symposium [1]:

(1) "All eubacteria that gain energy from photosynthesis or oxygen-respiration and are capable of haloadaptation, are able to accumulate and/or produce compatible solutes."

For the Bacteria that lead an oxygenic or anoxygenic phototrophic life this statement is still true. However, the discovery of *Salinibacter ruber*, phylogenetically affiliated with the *Bacteroidetes *but physiologically behaving like a member of the *Halobacteriaceae*, shows that the 'high-salt-in strategy' of osmotic adaptation, based on the accumulation of KCl and the adaptation of the entire intracellular enzymatic machinery to the presence of high salt concentrations, also occurs in some oxygen-respiring members of the domain Bacteria.

(2) "Archaeobacteria and anaerobic fermenting eubacteria are incapable of synthesizing organic compatible solutes."

No cases of anaerobic fermenting Bacteria that produce organic osmotic solutes have yet been documented. However, compatible solutes have been detected in several groups of halophilic Archaea. Methanogenic Archaea (also the non-halophilic ones) contain high concentrations of KCl, but in the halophilic types such as *Methanohalophilus *spp. different organic solutes are known to occur: glycine betaine, and β-amino acids and derivatives. The intracellular concentrations of at least some of these compounds are regulated in accordance with the salinity of the environment. Another case of organic solutes discovered in Archaea is that of sulfotrehalose which, together with KCl, appears to play a role in the osmotic adaptation of some haloalkaliphilic members of the *Halobacteriaceae*.

(3) "Most eubacteria that can grow in non-complex media are capable of ectoine biosynthesis."

Ectoine indeed appears to be the nearly universal organic compatible solutes in the Bacteria, and its biosynthesis has been documented in a wide variety of halophilic and halotolerant species, and notably in species with simple growth demands.

(4) "Extreme halophily in non-fermenting eubacteria is usually accompanied by glycine betaine biosynthesis. The complete de novo synthesis of betaine from carbon dioxide or simple organic carbon compounds has only been proven for cyanobacteria, *Ectothiorhodospira *species and *Actinopolyspora halophila*."

The truly halophilic species within the genus *Ectothiorhodospira *have since been reclassified in the newly created genus *Halorhodospira*. The finding of complete de novo biosynthesis of glycine betaine in the methanogenic archaeon *Methanohalophilus portucalensis *and in haloalkaliphilic bacterial chemolithotrophs shows that production of this simple solute may be more widespread than previously assumed.

Summarizing all these new data, it becomes ever more difficult to formulate general trends to describe the relations between halophily, taxonomy, phylogeny and nomenclature. The halophilic microbial world is tremendously diverse, and novel types of halophiles are being discovered at an ever-increasing rate. The case of *Salinibacter ruber *shows that some of these newly described organisms may necessitate us to revise our concepts on how the different physiological and phylogenetic groups of microorganisms have solved the problem how to cope with high salt concentrations in their environment. Hypersaline environments are extremely diverse, and so are the microorganisms that inhabit them.
